# Supplementary material for: A new rat model of creatine transporter deficiency reveals behavioral disorder and altered brain metabolism
Source: Sci Rep. 2021 Jan 15;11:1636. doi: 10.1038/s41598-020-80824-x (PMC7810893; doi:10.1038/s41598-020-80824-x)
Supplement: Supplementary file 1 — Supplementary Information [file 41598_2020_80824_MOESM1_ESM.docx]

**Supplementary materials**

**A new rat model of creatine transporter deficiency reveals**

**behavioral disorder and altered brain metabolism**

Lara Duran-Trio^1^, Gabriella Fernandes-Pires^1^, Dunja Simicic^2^, Jocelyn Grosse^3^,

Clothilde Roux-Petronelli^1^, Stephen J. Bruce^1^, Pierre-Alain Binz^1^, Carmen Sandi^3^,

Cristina Cudalbu^2^, Olivier Braissant^1^

^1^ Service of Clinical Chemistry, University of Lausanne and University Hospital of Lausanne, Lausanne, Switzerland

^2^ Centre d’Imagerie Biomedicale (CIBM), Ecole Polytechnique Fédérale de Lausanne (EPFL), Lausanne, Switzerland

^3^ Brain Mind Institute, Ecole Polytechnique Fédérale de Lausanne (EPFL), Lausanne, Switzerland

**Supplementary method :**

## *Generation, of the Slc6a8^Y389C^ KI rat strain*

The Y389C (TAC to TGC) mutation site in donor oligonucleotide was introduced into exon 8 by homology-directed repair. Cas9 mRNA, gRNA generated by in vitro transcription and donor oligonucleotide were co-injected into fertilized eggs for KI rat production. The pups were genotyped by PCR followed by sequence analysis, the positive ones being bred to the next generation and subsequently also verified by PCR and sequencing. 8 potential off-target sites were identified and verified by sequencing, all showing no mutations.

**Supplementary Figure 1 :**

***
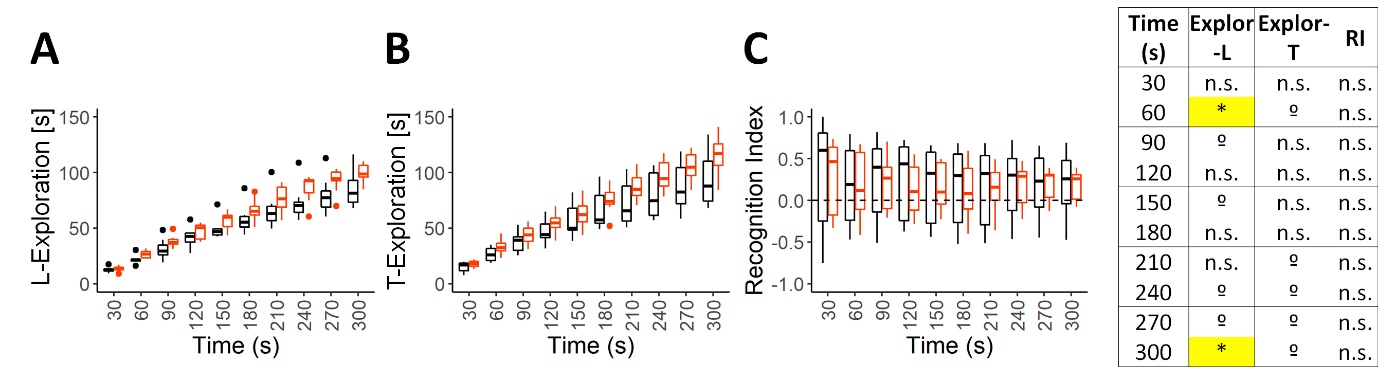
***

***Slc6a8^xY389C/y^* KI males did not present differences in NOR test (declarative memory).** (**A-B**) Boxplot graph showing the cumulative exploration time (in seconds) in each time-point (in blocks of 30 seconds) during the learning/retention phase (**A**, that is to say exploration of the two identical objects) and during the testing/recognition phase (**B**, that is to say the exploration of both new and familiar objects together). (**C**) Boxplot graph showing the recognition index (RI = ((Nobj-Fobj)/(Nobj+Fobj)), Nobj and Fobj being the cumulative exploration times of the novel and familiar objects respectively in each time-point along the 5 minutes total of the testing phase. The table shows the p-values between genotypes (two-tail t-test; *p<0.05; ºp<0.1; n.s. not significant) for each time point along the 5 minutes total for each variable measured: cumulative exploration time in the learning phase (Explor-L), in the testing phase (Explor-T), and the RI. Graphs were done using ggplot2 package ^29^.
